# Aging Skeletal Muscles: What Are the Mechanisms of Age-Related Loss of Strength and Muscle Mass, and Can We Impede Its Development and Progression?

**DOI:** 10.3390/ijms252010932

**Published:** 2024-10-11

**Authors:** Thomas Gustafsson, Brun Ulfhake

**Affiliations:** Department of Laboratory Medicine, Karolinska Institutet, 171 77 Stockholm, Sweden; thomas.gustafsson@ki.se

**Keywords:** dynapenia, muscle fibre atrophy, motor unit, ageing, senescence

## Abstract

As we age, we lose muscle strength and power, a condition commonly referred to as sarcopenia (ICD-10-CM code (M62.84)). The prevalence of sarcopenia is about 5–10% of the elderly population, resulting in varying degrees of disability. In this review we emphasise that sarcopenia does not occur suddenly. It is an aging-induced deterioration that occurs over time and is only recognised as a disease when it manifests clinically in the 6th–7th decade of life. Evidence from animal studies, elite athletes and longitudinal population studies all confirms that the underlying process has been ongoing for decades once sarcopenia has manifested. We present hypotheses about the mechanism(s) underlying this process and their supporting evidence. We briefly review various proposals to impede sarcopenia, including cell therapy, reducing senescent cells and their secretome, utilising targets revealed by the skeletal muscle secretome, and muscle innervation. We conclude that although there are potential candidates and ongoing preclinical and clinical trials with drug treatments, the only evidence-based intervention today for humans is exercise. We present different exercise programmes and discuss to what extent the interindividual susceptibility to developing sarcopenia is due to our genetic predisposition or lifestyle factors.

## 1. Popular Summary

In this review, we present the current evidence on the background to age-related muscle weakness (sarcopenia), which affects around 5–10% of older people in society, and how we can help intervene in the disease process to improve the quality of life of those affected. We will discuss what is currently known about the cause of this disease. Is it due to age-related changes in the muscle fibres, the muscle organ or the innervation of the muscle? We showcase efforts to impede sarcopenia by restoring a more youthful skeletal musculature and the drug treatments currently being tested in experimental models and clinical trials. However, we conclude that to date there is no biomedical treatment that can halt the condition, and that the only evidence-based intervention for humans is physical exercise. Finally, we discuss whether the large differences between people in the severity of sarcopenia depend on their predisposition or lifestyle.

## 2. Growing Old

In the last two centuries, life expectancy at birth has almost doubled, while years in health have not improved to the same extent (discussed in [1]). It is widely believed that the deterioration in health at older ages is due to the cumulative detrimental effects of aging at subcellular, cellular, systemic and organismal levels. The challenge we now face is therefore to extend the healthy lifespan through evidence-based lifestyle recommendations and rational biomedical interventions.

Aging is accompanied by the characteristic phenotypic changes with which we are all familiar. These include loss of connective tissue of integrity, which leads to a decrease in lung compliance and stiffening of blood vessels, which affects blood pressure and cardiac workload [2,3,4,5]. In the musculoskeletal system, there is a loss of bone (osteopenia and osteoporosis), joint cartilage and skeletal muscle function and mass (sarcopenia) [6,7]. These changes hinder movement and balance and drastically increase the risk of falls and bone fractures. At the same time, there is an accumulation of senescent cells and undegraded waste (e.g., lipofuscin). These changes do not occur suddenly. They begin slowly and accelerate with age, but are not closely linked to chronological age. The genetic predisposition of the individual in combination with random and fixed environmental factors is reflected in a considerable variability in the biological age of people of the same chronological age [8,9] (discussed in [1]). The search for markers of biological age is currently attracting considerable attention, but has not yet produced reliable and validated markers for use in humans [10,11,12].

The decline in function and structural integrity of the aged phenotype is intrinsically driven by two main processes: the failure to replace senescent cells (reviewed in [13]), and an inadequate machinery to maintain cellular and extracellular homeostasis (reviewed in [1,14,15]).

## 3. Skeletal Muscle and Its Innervation

The power to balance and move our bodies is generated by the contraction (tension) of the myofibres in skeletal muscles, and the size of a healthy skeletal muscle reflects the number of myofibres it contains. Myofibres are highly specialised multinucleated structures with a cross-sectional diameter of ~0.1 mm and a length that varies from about one centimetre to several tens of centimetres. A myofibre is anchored at both ends by connective tissue that connects the muscle to the skeleton. The collective tension of all active myofibres in a muscle creates a torque at the joints between the bones of our skeleton, and this force allows us to balance gravity and move. Normally it is the central nervous system that controls the activity of our muscles. Using sensory information about position and muscle tension, the central nervous system circuits coordinate a motor output to produce, for example, a reflex or a movement pattern. More specifically, the motor neurons (MNs) in the spinal cord and brainstem of the central nervous system trigger the contraction of skeletal muscle fibres by sending signals that are transmitted through the neuromuscular junctions (NMJs). An NMJ is a synapse, formed by the interaction of the innervating motor axon terminal, adjoining Schwann cells and the endplate (apposing area) of the myolemma. After development a myofibre has only one innervation site, i.e., one NMJ (for a more detailed account of the NMJ, see [16,17].

Each motor neuron innervates a number of myofibres (between ~8 and >1000 depending on muscle type and type of motor unit), which together form a motor unit (MU) [18]. All myofibres of an MU are of the same type: slow (type I; S-type MU) in contraction speed (twitch time) or fast contracting (type II), and the latter type of MU (F-type MU) often contains many more myofibres than the slow type [19,20]. The MUs of a muscle are the building blocks of graded muscle contraction (idem). The main mechanism by which we regulate muscle strength is therefore the recruitment and de-recruitment of MUs [20,21]. The timing and precision of these processes are crucial for all body movements and for balancing the pull of gravity acting on our body mass. A second mechanism by which we can modulate muscle tension is by increasing the MN firing frequency (up to the fusion rate) until a maximum is reached (~×1.5 in tension; Tetani) (see e.g., [22]. This mechanism depends on the firing characteristics and impulse conduction properties of the MNs (idem).

Skeletal muscle is an organ with an internal structure that supports its functions and adaptation to changing demands. The connective tissue provides the structure and the cellular and molecular environment (scaffold, stroma) for the myofibres and their stem cell niche as well as pathways for the vasculature and the innervation of the muscle organ (see below, and for a detailed account see [2,5,23,24,25,26,27,28,29]).

### 3.1. Loosing Muscle Strength and Mass

**Figure 1:**
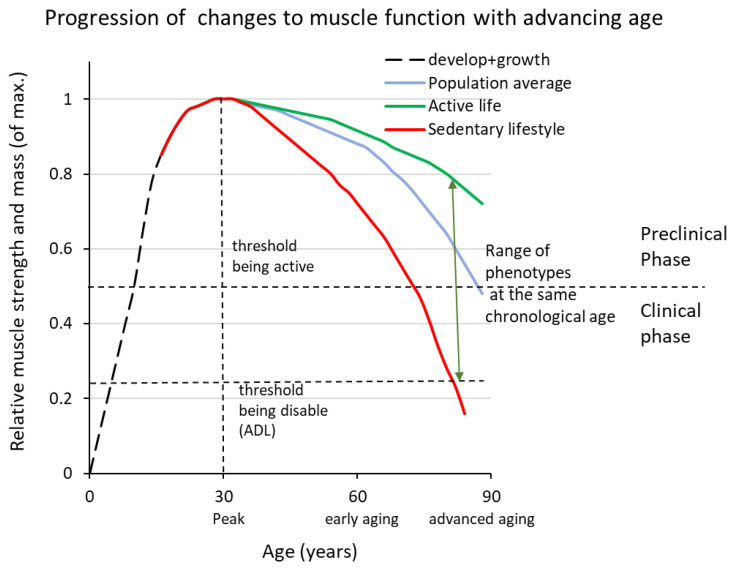
Shows a schematic representation of the lifespan trajectory of changes in muscle strength and mass. The age at which peak performance is reached depends on the type of activity but is usually in the range of 25–35 years of age (dashed vertical line). Three curves are shown, representing very physically active individuals (green line), a person with 2–4 h of leisure-time physical activity per week (blue line) and a person with a sedentary lifestyle (red line). What all three phenotypes have in common is that peak performance is reached at around 25–30 years of age and that the rate of loss is initially slow but accelerates with advancing age. The preclinical phase continues when daily activities are not affected, while the need to moderate physical activities to cope with the loss of muscle mass and strength marks the entry into the clinical phase (the upper horizontal dashed line). As the process progresses, it eventually leads to disability (lower horizontal dashed line). Note that the range of phenotypes in the population is the product of genotype and lifestyle.

As we enter middle age and beyond, most of us find that the speed of muscle contraction, strength and mass decline with age. When the loss of strength and mass reaches a threshold (Figure 1) it is referred to as sarcopenia (definition guidelines for diagnostic criteria have been published and revised by European, Asian and North American working groups (the EWGSOP2 [30], the Asian Working Group for Sarcopenia (AWGS) [31], and the FNIH [32]), a condition that becomes clinically significant in the sixth decade of life with an annual increase of 0.5–2% in the elderly population. In advanced age, sarcopenia can be the main or sole cause of disability in daily life and has its own disease code (WHO [33]; ICD-10-CM code (M62.84) [34]). The prevalence of sarcopenia in older people varies from population to population but is generally in the range of 5–10%. Here we use the term sarcopenia in its broader definition and do not refer to dynapenia (loss of strength) as a separate entity.

The decline in muscle strength and contraction speed (velocity) precedes the loss of muscle mass (for references see [1]) and these are changes that are already detectable beyond the age of 30 in elite athletes [35,36,37,38,39] and in population-wide cohorts [40] (Figure 1). Initially, the rate of decline is slow (<1% per year), but later the loss accelerates (~1–2% per year), and by the time untrained individuals notice it, it has already lasted for decades (idem). The rate of decline is usually expressed as a percentage change per year from a given start and end point, whereas a better assessment of rate would be the change in strength and mass between successive measurements of a longitudinal time series of measurements.

Below we will refer to the early phase as the preclinical phase, while the overt condition is referred to as the clinical phase of sarcopenia (Figure 1; [41]).

There is still a lack of both evidence and scientific consensus on the mechanism(s) underlying the loss of muscle strength and mass that can lead to sarcopenia (for references see [1,6,7,16,17,42]. Below is a brief description of the aging of the neuromuscular system with references to more detailed accounts.

### 3.2. Aging of Motor Units

**Figure 2:**
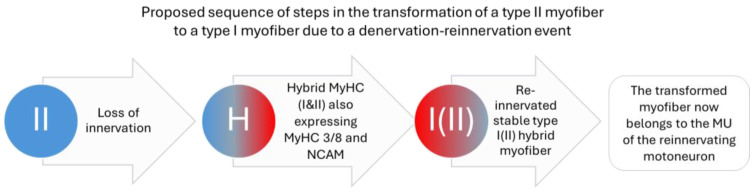
In this schematic example, a fast myofibre expressing type II myosin is denervated due to axon atrophy. In the denervated state, fast (and slow) myofibres often express both type I and type II myosins, plus embryonic myosin as a sign of denervation. When the fast myofibre is reinnervated by a slow MN, it switches to type I myosin as the predominant form, while residual amounts of type II myosin can often be detected. However, the expression of embryonic myosin is suppressed. We refer to this phenotype as hybrid myofibre. During aging, the frequency of hybrid myofibres increases from <1% to several percent of the myofibre population.

Recordings from humans and animal models show that the number of MUs per muscle decreases continuously during aging and that the surviving MUs increase in size in parallel. The interpretation of these observations is that myofibres are denervated due to the degeneration of motor axons and, in parallel, some of the denervated myofibres are re-innervated by nearby intact motor axons (increase in MU size) (Figure 2) [7,16,17,43,44,45], the number of denervated fibres will increase with age, as the capacity for re-innervation is limited (idem).

Observations in the peripheral and central nervous system have shown that axonal degeneration with atrophy and dystrophy—in both cases the axon terminals at synaptic contacts are lost—is widespread and more conspicuous than the loss of neurons during aging (reviewed in [46]). This process is referred to as “dying-back” of neurons, and projecting neurons, such as MNs, appear to be more susceptible than short circuit neurons (idem). In addition to the MNs themselves, the affected systems include the descending bulbospinal aminergic systems, which act as gain-setters for MN excitability [47,48,49,50,51,52]. Thus, MN innervation of myofibres is vulnerable and impaired during aging, and the available evidence also supports the notion that age-related degeneration of some of the gain-setting systems of MN excitability in the nervous system may affect the firing probability and firing frequency of aged MNs [53,54].

It is not yet clear whether the loss of MUs during aging is solely due to the inability to maintain innervation by the motoneurons, or whether this process is also driven by changes in the target myofibres and/or the local environment (muscle scaffold), or a combination of both. NMJs exhibit a number of molecular and structural changes including removal of pre-terminal axons and a disintegration (fragmentation) of myofibre endplates (reviewed in [7,16,17,45]. However, if we consider that many denervated myofibres are successfully re-innervated since the loss of MUs is accompanied by an increase in the size of the remaining MUs, it seems reasonable that at least the early phase of sarcopenia has a neuronal origin. In later stages, changes in the local environment and the target myofibres could play an important role. The interplay between these factors should be investigated further. It also remains unresolved if the transmission across the NMJ is impaired prior to loss of innervation, as recordings of the transmission at NMJ during aging show conflicting results (reviewed in [17]). Another unresolved question is why the loss of MU preferentially affects the fast-twitch MUs, while the slow-twitch MUs increase in size. These two questions need to be clarified.

Importantly, aging-induced remodelling of a muscle’s MU population also affects individuals who train at a very high level throughout their lives [55,56,57,58,59]. The remodelling of the MU population leads to a general slowdown in motor behaviour and a decrease in muscle strength and power output. In the early stages, total muscle mass is not affected to the same extent, whereas in advanced age, mass also decreases (Figure 1).

**Figure 3:**
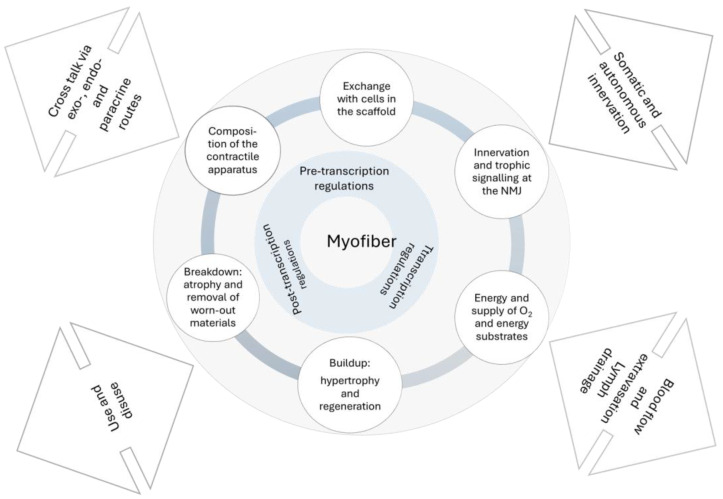
Schematic representation of the myofibre’s inherent tools that enable adaptive and regenerative responses. The interface with the tissues involved in balance and movement processes and the system level are indicated by bidirectional arrows.

Microscopic observations in aged sarcopenic muscles show an increased variability in myofibre size and clusters of severely atrophied fibres, and an increase in interstitial tissue (fibrosis see below). In advanced age, the adaptive response (hypertrophy) of myofibres to exercise is attenuated, a change attributed to impaired anabolic response and impaired recruitment of myocytes from the local stem cell pool (satellite cells; SC, niche) (Figure 3; discussed in [1,13]). Aged myofibres exhibit increased lipid content and dysfunctional mitochondria and there are signs of impaired autophagy-lysosomal and proteasomal degradation of worn-out cellular components with an accumulation of polyubiquitinated proteins and lipofuscin (Figure 3; [60,61]).

The replacement and maintenance mechanisms described above are crucial for the adaptive and regenerative capacity of skeletal muscles. While MNs rely solely on maintaining their functionality with age, skeletal muscle is also able to adapt and regenerate by increasing fibre size through anabolism and the recruitment of locally available stem cells (satellite cells, SC). However, with age, the size of the pool of progenitor cells (SC) decreases and many of the remaining SC do not respond to stimuli to proliferate and supply the myofibre with additional myonuclei [62]. Furthermore, the intrinsic machineries for anabolism and catabolism in myofibres appear to be dysregulated. Thus, despite the myofibre atrophy seen in aged muscles, the level of the key scaffold that drives myofibre hypertrophy (mTORC1) is increased and not decreased [63,64]; similarly, the main machinery for degradation of targeted proteins (through proteasomal degradation) is also increased but dysregulated [60,61]. These disruptions in the resources and machinery crucial for myofibre adaptations may explain why aged myofibres show poorer or blunted adaptive responses to stimuli such as exercise (see also below).

Myofibres also secrete molecules (secretomes; e.g., chemokines, myokines and trophic factors) through which they communicate not only with neighbouring myofibres, SCs and axons of peripheral motor nerves, but also with the immune system, bone tissue, and cells of the muscle stroma including resident macrophages [65,66,67,68,69]. The secretome aroused great interest in the search for useful target molecules to impede the sarcopenic process (see below), especially those secreted after exercise (exerkines; idem).

### 3.3. Aging of the Muscle Scaffold

In sarcopenia research, much less attention has been paid to the scaffold than to the myofibres and their innervation. The scaffold is colonised by stromal cells derived from fibro-adipogenic mesenchymal progenitor cells [2,5,23,24,25,26,27,28,29]. In skeletal muscle, it is mesodermal fibroblasts that are adapted to the skeletal muscle organ and produce the ECM, which consists of proteins. The proteins form fibrils by cross-linking, and fibrils combine to form fibres with a complex 3D structure that can grow into sheaths (fascial structures). These divide a skeletal muscle into tensile compartments, in which the sarcolemma of a single myofibre is surrounded by a bilayered basal lamina (endomysium), groups of muscle fibres are separated by septal sheaths (perimysium) and the entire muscle is enveloped by the muscle fascia (epimysium). The ECM of these units has a distinctly different composition, reflecting their respective function (idem). At one or both ends of a muscle, the ECM connects the muscle to a tendon. The tendon anchors the muscle to the skeleton. The composition of the ECM of a tendon differs from the ECM of the muscle and is formed by tenoblasts of mesenchymal origin [70,71]. Together, the ECM of the muscle and the tendon transmit the tension generated by the contraction of the myofibres to the bone. It is the structure of the muscle ECM that allows the myofibres in multi-pennate muscles to pull in concert on the tendon.

Molecules secreted by fibroblasts (secretome, chemokines, myokines and trophic factors) communicate not only with myofibres, SCs and axons of peripheral motor nerves, but also with the immune system and resident macrophages [5,72] and bone tissue (bone–muscle crosstalk [67,73]). ECM remodelling occurs slowly and is controlled by the resident fibroblasts under normal conditions. Fibroblasts produce both the ECM and the proteases that digest it (mainly matrix metalloproteases MMPs) [5,29]. As ECM proteins are long-lived, they are targets for environmental induced secondary modifications such as glycosylation. Such adducts can impair the proper function of the ECM [24].

With increasing age, changes (fibrosis) occur in the ECM, leading to increased stiffness and fragility of the tendon and muscle compartments [23,24,26,74]. The changes to the molecular and cellular components of the scaffold are thought to contribute to the age-related impairment of myocyte renewal from SCs. Changes in the ECM could play a role in the failure to reinnervate denervated myofibres by collateral sprouting in advanced age. Fibrosis in sarcopenic muscles indicates an imbalance in the production, (re)assembly and degradation of ECM components [23,75,76]. In summary, the muscle scaffold is extensively affected in parallel with the changes in the myofibres during aging and may play an important role in the progression towards sarcopenia.

## 4. Cellular Senescence

Cellular senescence is a term introduced half a century ago to describe the loss of the ability of cells to replicate that occurs in cell cultures. Such cells no longer respond to environmental stimuli and therefore no longer contribute to tissue function in vivo [76,77]. More recently, it has been found that the number of senescent cells increases with age and that they become a burden to the tissue due to their space requirements and—perhaps more importantly—their secretome, referred to as SAPS (senescence associated protein secretory (cell) phenotype) [76,78,79]. It has been hypothesized that the age-related accumulation of senescent cells is due to an increase in the rate at which cells become senescent and/or a decrease in the ability of the immune system’s natural killer (NK) cells to remove these cells [80]. One attempt to improve the regenerative and adaptive capacity of tissues in old age is therefore to find an intervention that promotes the removal of senescent cells (idem).

## 5. Interventions to Impede Sarcopenia

If we do not use our muscles in everyday life, we lose strength and muscle mass [81]. Not even top athletes who train at a high level throughout their lives are spared the age-related decline in muscle function. In fact, the data available today does not show that the rate of decline differs between elite athletes, recreational athletes and people who lead a more sedentary life. However, people who exercise regularly are more successful at maintaining their mobility and independence in old age than those who lead sedentary lives [39,82] (for a more detailed discussion on this topic, see [1,83,84,85]). The loss of muscle strength and mass due to disuse is not only due to a sedentary lifestyle. In older age, recurrent hospitalizations and, for example, bone fractures due to falls lead to repeated episodes of muscle deconditioning, which could accelerate the progression of sarcopenia. In experimental models of muscle disuse (unloading) in young adult humans, the loss of strength is greater than the reduction in muscle mass and is accompanied by changes in the innervation detected by EMG [86,87]. However, these changes are reversible at least in adulthood and are driven by muscle unloading, whereas the loss of muscle strength and mass during aging is not reversible and occurs despite intensive muscle activity. Muscle disuse should therefore be considered as a factor that accelerates the progression from preclinical to clinical sarcopenia.

The prevalence of sarcopenia in older people is 5–10% of the elderly population and leads to varying degrees of disability. In older people, poor diet and malnutrition, comorbidities and physical inactivity have been identified as major factors in the progression of preclinical to clinical sarcopenia, requiring a multimodal intervention approach to improve nutrition, increase physical activity and adjuvant biomedical treatment [88,89,90,91,92]. With regard to nutrition and diet, we refer to the work cited above and will explain in more detail below that exercise enables us to utilise the adaptive potential of the neuromuscular system and connective tissue as we age.

In an effort to counteract the decline in adaptive potential of myofibres with age and also to address situations where exercise is less feasible or an insufficient intervention, a number of biomedical treatment strategies have evolved, including cell-based and drug treatments, as well as senotherapies [13,93,94,95]. These therapeutics are still mainly in the experimental preclinical stage of development and our current understanding of the underlying mechanisms of such treatments and those underlying sarcopenia is mainly based on animal models and ex vivo research.

### 5.1. Exercise

The only evidence-based intervention to combat the decline in muscle function and mass is exercise, and this also applies to frail individuals suffering from multiple age-related comorbidities. The uptake of exercise in older age is not without its challenges and needs to be individualized to optimize the cost-benefit ratio. For untrained, frail individuals, balance and flexibility of the body should be trained first and then regular endurance and strength exercises should be introduced. Even at low intensity and low volume (e.g., once a week), most people make progress within a few weeks, initially through improved postural and neuromuscular control, and later, depending on the type and dose of training, also in endurance, strength and muscle mass [96,97,98]. In advanced age, it is perhaps most important to avoid periods of detraining to minimize deconditioning of neuromuscular control and muscle mass [99]. As adaptive capacity decreases with age, it becomes increasingly difficult to recover from periods of immobilization (deconditioning). Such episodes should be kept as short as possible and supplemented by a re-training program [1]. In recent decades there has been a lively debate about the relative benefits of different forms of exercise (endurance training vs. strength training), the intensity and frequency of training and the amount of exercise. Endurance training (aerobic exercise) (ET) improves cardiovascular function and oxygen uptake (VO_2_), cellular respiration (mitochondrial function and mtDNA copy number), muscle capillarisation and the ECM, but has a lesser effect on overall muscle strength and mass [75,85,100,101,102,103,104]. In addition, animal models have shown that regular exercise protects the integrity of the NMJ during aging [105].

The multisystemic effect of ET counteracts several of the observed consequences of aging, such as mitochondrial dysfunction, low-level inflammation, reduced aerobic capacity (cardiovascular and pulmonary system), and changes in connective tissue (e.g., stiffening of the arteries and cardiac and pulmonary compliance as well as fibrosis of the muscle scaffold). Therefore, lifelong ET is a measure to counteract the consequences of aging on a systemic level [4,106,107]. Regular endurance training is less effective in preventing the loss of muscle strength and mass in advanced age, and ET used as a mobilization measure in older people does not lead to a significant increase in muscle mass or strength [108].

Resistance training (RT) is an effective means of improving strength and muscle mass [82,96,97,98,109,110]. The response to RT in terms of improving strength and myofibre hypertrophy depends on the duration, frequency and intensity of training, with the current prevailing opinion being the more, the better. However, high intensity and very large amounts of RT may be counterproductive in older and frail individuals [98,110]. Although strength increases with RT dose, the response of myofibres to hypertrophy is lower and sometimes blunted in the elderly, indicating impaired myofibre adaptive potential (see above and [1,111,112]. Therefore, in older people, improvement in strength rather than muscle mass is a better measure of the success of RT. From the above, it seems logical that exercise programs to combat the onset and progression of sarcopenia should be based on both ET and RT, as they have complementary effects on the aging process, and a large number of studies point to the dual benefits of concurrent ET and RT [100,113,114,115,116,117,118,119].

In conclusion, simultaneous training of ET and RT is currently the best measure to slow the progression of sarcopenia and physical disability in older people of both sexes, while physical inactivity (unloading) accelerates the loss of strength and muscle mass. Although lifelong training is preferable, it is recommended to start training even in frail sedentary older people to slow down the progressive deconditioning of skeletal muscles. Especially in the frail elderly, these efforts should begin with balance, flexibility and coordination training, followed by strength and endurance training, with all components adapted to the subject’s situation. Even with a low frequency, such as one session per week, most people will see improvements. There exist guidelines on recommended physical activity, such as “The 2018 physical activity guidelines for Americans”, and studies have shown that those who adhere to the guidelines’ recommendations have a significant survival advantage [120]. The frequency and intensity of RT and ET sessions can be continuously adjusted as progress is made. Three sessions per week (totalling ~150 min at a moderate level) is a generally recommended value for repeated training. Overtraining should be avoided, while targeted recovery training after episodes of immobilization is strongly recommended. Balance, coordination, endurance, muscle strength and power are useful measures, while muscle mass/myofibre size is a less appropriate measure in advanced age.

### 5.2. Biomedical Interventions

Although exercise is an effective measure to slow down sarcopenia, it does not eliminate it and there are situations where exercise is not enough. With prolonged or repeated deconditioning, recovery may be inadequate, leading to physical disability and rapid progression of sarcopenia. Therefore, strategies to rejuvenate the adaptive potential of myofibres and mimetics that could replace or enhance the effect of ET/RT sessions to bridge episodes of immobilisation are being intensively researched, but have not yet led to a breakthrough in clinical practice. These strategies include stem cell transplantation, adjuvant endocrine therapy, senotherapies against senescent cells burden and their SAPs, and the use of molecules secreted by exercised myofibres (secretome) [62,69,93,94,121,122,123,124,125,126].

Rejuvenation of adaptive regeneration capacity by reconstitution of SC cells has not been successful so far (discussed in [13]). Rejuvenation of SC in vitro for subsequent autologous re-transplantation could be an alternative, but has not yet been tested in humans [123].

Another approach is to reduce the burden of senescent cells with senolytic drugs and their SAPS with senomorphic drugs. Here, trials with tyrosine kinase inhibitors such as dastinib (a drug already used to treat certain cancers) and quercetin or fisetin show promising results in animal models, but have yet to be tested in humans ([76,78,81,127]). Quercetin, fisetin and resveratrol are natural (poly)phenols, molecules that act as antioxidants and activate sirtuins, a family of proteins (SIRT1 to SIRT6) associated with the promotion of longevity [93,95]; for references see also [1].

Another strategy in this context is the use of neutralising antibodies against circulating levels of interleukin IL-6 [76], which is thought to be responsible for the low-level tissue inflammation that is a known component of the ageing phenotype; or antibodies against myostatin [128], a prominent trigger of myofibre atrophy via the TGFβ-Activin-Smad pathway. Both IL-6 and myostatin are components of the myofibre secretome [68]. However, this strategy is unlikely to be applicable to large numbers of people or over a prolonged period of time. Furthermore, data from experimental studies suggest that IL-6 secreted by myofibres plays an important role in enhancing the myotrophic response to exercise [129,130].

The secretome of myofibres is very diverse and more than 600 proteins have been identified to date. The content of the secretome is context-dependent [66,68] and at least 30 proteins are induced by exercise, including irisin, S100 (A and/or B) and apelin. Apelin, a secreted myokine that acts on the g-protein-coupled APJ receptor, has attracted considerable attention as a potential mediator of myofibre adaptation to exercise [69,122,131,132,133,134], and clinical trials may follow.

In this context, it is interesting to note that apelin protects the brain from cytotoxic damage by inducing Brain-Derived Neurotrophic Factor (BDNF) [135]. BDNF belongs to the family of neurotrophins (NTs), another group of proteins that increase in myofibres in response to exercise [124,136]. NTs are of interest not only because they play an important role during development and possibly also in the regeneration of myofibres due to their effect on SCs, but also because they support motoneurons and show characteristic expression changes during aging [43,125,137]. For example, satellite cells of the extraocular eye muscles, muscles that escape age-related loss of function and mass, express higher levels of NTs and their receptors compared to limb muscles [138]. However, how NT signalling in the muscles can be promoted during aging, apart from training, remains to be clarified.

Endocrine therapy with growth hormones, gonadal steroids or androgens has been widely discussed and tested in humans, but the risks associated with these molecules may outweigh the benefits of their use.

Efforts to preserve muscles through shorter periods of unloading with partial mimetics of caloric restriction (CR; the gold standard for slowing aging; discussed in [1,139,140]) such as metformin (a drug used to treat type II diabetes) in humans and rapamycin-like molecules (inhibitor of the mTORC1 complex) in animal models appear to be promising [63,141].

In summary, research efforts to date have not been successful in developing a biomedical treatment to preserve neuromuscular function during inactivity or ageing.

## 6. Concluding Remarks

The trajectory of physical performance in adulthood, with a peak at around 30 years of age, followed by a decline that is initially slow but aggravates with age, appears to be universal for humans and suggests that the trajectory is constitutive and not acquired. Furthermore, there are no data available to help us understand the extent to which the interindividual differences in the progression (rate of decline) and extent of sarcopenia are due to genetic variation. In general, it is estimated that heredity accounts for only 25% of the variability in the human lifespan (see above). Data on muscle mass and strength show a greater influence of heredity, estimated at ≥50% [142,143,144]. However, cross-generational studies on the relationship between genetic variations and the development and progression of sarcopenia are lacking, but are extremely valuable for developing future interventions at an individual level.

To further our knowledge of the mechanisms underlying sarcopenia, we need longitudinal observations that identify the turning point at which the number of MU and the speed and force of MU contraction begin to decline, as well as the events in the nervous system, myofibres, and muscle scaffold (ECM) that precede this moment. Therefore, we should not focus on those who already have manifest or incipient sarcopenia, but on adults who are about to pass the peak of their physical performance. In the later stages of sarcopenia progression, it is difficult to distinguish cause from consequence. As has already been emphasised, myofibres and their innervation depend on a well-maintained ECM to preserve their adaptive and regenerative capacity. The ECM has not received the same attention as myofibres, myofibre innervation and resident myocyte precursors, i.e., SCs. Simultaneous consideration of the ECM in future studies on sarcopenia is urgently needed.

Similar mechanisms may be active in the motor neurons and myofibres as well as in the cells of the muscle scaffold. The timing of events and speed of progression between the different cell and tissue types may be due to differences in their susceptibility rather than differences in the driving mechanisms. A common mechanism is suggested by the fact that the universal gold standard for delaying the onset of ageing in all physiological systems of an organism is caloric (dietary) restriction (CR; [145]). As discussed elsewhere [139], CR is not a realistic intervention in humans, but it can help us find molecules that act as CR mimetics.
